# Conditional knock-out of lipoic acid protein ligase 1 reveals redundancy pathway for lipoic acid metabolism in *Plasmodium berghei* malaria parasite

**DOI:** 10.1186/s13071-017-2253-y

**Published:** 2017-06-27

**Authors:** Min Wang, Qiong Wang, Xiang Gao, Zhong Su

**Affiliations:** 1 University of Science and Technology of China, Fei Xi Road, Hefei, 230000 China; 2 Laboratory of Immunobiology, Guangzhou Institute of Biomedicine and Health, Chinese Academy of Sciences, 190 Kai Yuan Road, Guangzhou, 510530 China

**Keywords:** *Plasmodium berghei*, Lipoic acid protein ligase, Tetracycline-inducible transcription system, Conditional knockout

## Abstract

**Background:**

Lipoic acid is a cofactor for α-keto acid dehydrogenase system that is involved in the central energy metabolism. In the apicomplexan parasite, *Plasmodium*, lipoic acid protein ligase 1 (LplA1) and LplA2 catalyse the ligation of acquired lipoic acid to the dehydrogenase complexes in the mitochondrion. The enzymes LipB and LipA mediate lipoic acid synthesis and ligation to the enzymes in the apicoplast. These enzymes in the lipoic acid metabolism machinery have been shown to play important roles in the biology of *Plasmodium* parasites, but the relationship between the enzymes is not fully elucidated.

**Methods:**

We used an anhydrotetracycline (ATc)-inducible transcription system to generate transgenic *P. berghei* parasites in which the *lplA1* gene was conditionally knocked out (LplA1-cKO). Phenotypic changes and the *lplA1* and *lplA2* gene expression profiles of cloned LplA1-cKO parasites were analysed.

**Results:**

LplA1-cKO parasites showed severely impaired growth in vivo in the first 8 days of infection, and retarded blood-stage development in vitro, in the absence of ATc. However, these parasites resumed viability in the late stage of infection and mounted high levels of parasitemia leading to the death of the hosts. Although *lplA1* mRNA expression was regulated tightly by ATc during the whole course of infection, *lplA2* mRNA expression was significantly increased in the late stage of infection only in the LplA1-cKO parasites that were not exposed to ATc.

**Conclusions:**

The *lplA2* gene can be activated as an alternative pathway to compensate for the loss of LplA1 activity and to maintain lipoic acid metabolism.

**Electronic supplementary material:**

The online version of this article (doi:10.1186/s13071-017-2253-y) contains supplementary material, which is available to authorized users.

## Background

Lipoic acid (LA, 6,8-thiooctanoic acid) is a cofactor required for the function of α-keto acid dehydrogenases (KADH) and the glycine cleavage system (GCS). In most organisms, these enzyme complexes are present in the mitochondrion and involved in fatty acid biosynthesis, energy metabolism and amino acid degradation [1–4]. The cofactor LA that is ligated to the enzyme protein is supplied either by a biosynthesis pathway or by salvage from the environment. For LA biosynthesis, the octanoyl-acyl carrier protein (ACP) is first ligated to the E2-subunit of the KADHs and apo-H-protein of GCS under the catalysis by octanoyl-acyl carrier protein: protein N-octanoyl transferase (lipoic acid protein ligase B, LipB) [5]. It is followed by the insertion of two sulphurs at position C6 and C8 of the octanoyl moiety by lipoic acid synthase (LipA) to form the lipoyl-arm required for KADH and GCS activity [6, 7]. Alternatively, LA obtained from the environment is transferred to the E2-subunit of KADHs and apo-H-protein of GCS that is catalysed by two enzymes, lipoate activating enzyme and lipoyltransferase [8–10]. *Plasmodium* parasites, apicomplexan protozoan pathogens that cause malaria, also possesses a plastid-like organelle called the apicoplast that contains the LA-requiring enzyme machinery [11, 12]. It is now known that in *Plasmodium* parasites, the LA biosynthesis pathway and lipoylation of pyruvate dehydrogenase (PDH) only occurs in the apicoplast, while lipoylation of two other KADHs, α-ketoglutarate dehydrogenase (KGDH) and branched chain keto acid dehydrogenase (BCDH) as well as H-protein of GCS, by salvaged LA takes place in the mitochondrion [13–16]. Unlike the situation in mammals, salvaged LA is ligated to an E2-subuint of the enzyme complexes by lipoic acid protein ligase A (LplA) [17, 18]. Two forms of LplA have been identified in *Plasmodium* parasites with LplA1 being localised in the mitochondrion and LplA2 being present in both mitochondrion and the apicoplast [15, 19]. The LA metabolism pathways in *Plasmodium* parasites that is distinct from that of the mammalian host make them promising targets for the development of a malaria intervention strategy.

The previous study by Dahl et al. [20] showed that treatment of *P. falciparum* with antibiotic induced loss of apicoplast function and resulted in the delayed death of the parasites, suggesting that this organelle is required for parasite survival. Disruption of the lipB gene did not affect the growth of *P. falciparum* although the LA level was significantly reduced. Further analysis showed that LplA2 compensated for the loss of LipB activity and lipolyation of PDH in apicoplast [19]. Furthermore, lipoic acid analogue 8-bromoocttanoate inhibited the activity of LplA1, blocked the salvage of LA and arrested the growth of *P. falciparum* in vitro [18]. Günther et al. [21] reported that the *lplA1* gene in murine malaria parasite *P. berghei* can be targeted by cross-over recombination, but the *lplA1* knockout parasite population was not possible to isolate, indicating that this gene is essential for the survival of the parasite. These observations demonstrate that the LA metabolism machinery is critical for the survival and development of malaria parasites.

In this study, we used an anhydrotetracycline (ATC)-inducible gene expression system to conditionally knockout the *lplA1* (LplA1-cKO) gene in blood-stage *P. berghei* and analysed the phenotypical changes of the transgenic parasite. We observed that although the LplA1-cKO parasites showed arrested proliferation in vivo in the initial stage of infection in the absence of ATC, the transgenic parasites restored the viability in the late stage of infection. Further analysis revealed that *lplA2* gene expression was increased during the late stage of infection, suggesting a compensatory role of LplA2 for the loss of LplA1 activity.

## Methods

### Mice and parasites

The ANKA strain of *P. berghei* was obtained from BEI Resources Repository (NIH, Bethesda, MD, USA), propagated in BALB/c mice and stored in liquid nitrogen. Female BALB/c mice (6–8 weeks of age) was purchased from Vital River Laboratories (Beijing, China). All mice were housed in a specific-pathogen-free barrier facility. The blood stage infection of *P. berghei* was initiated by i.p. injection of 1 × 10^6^ parasitized red blood cells (pRBCs), and the parasitemia was monitored daily by examination of Giemsa-stained (Sigma-Aldrich, MO, USA) thin smears of tail blood.

### Construction of ATc-inducible gene expression vector

The tetracycline repressor (TetR) protein and tetracycline operon (TetO) sequence originally identified in the tetracycline (Tet) resistant *Escherichia coli*, were modified for regulation of gene expression in mammalian cells [22, 23]. We utilised these regulatory and responsive elements to generate an ATc-regulatable gene expression system for the blood-stage *P. berghei* parasite. Transfection vector was constructed using the plasmid pL0016 (BEI Resources Repository), that contains dihydrofolate reductase-thymidylate synthase derived from pyrimethamine-resistant *Toxoplasma gondii* (tgdhfr/ts) as a selectable marker, was originally designed for *P. berghei* [24]. This plasmid contains the green fluorescence protein (*gfp*) gene under the control of elongation factor-1 alpha promoter in *P. berghei* (PbEF1) [25]. The 657 nucleotide sequence encoding TetR was cloned using the primer pair TetR-1/TetR-657 (Additional file 1: Table S1) from the genomic DNA of the T-REx293 cell line that was transfected with pcDNA™6/TR (Invitrogen, Carlsbad, USA) [23] and inserted into pL0016 to replace the *gfp*, resulting in plasmid pL0016-TetR. The 3’UTR of *lplA1* of *P.berghei* (*PblplA1*) amplified using primers 3’A41/3’A42 was cloned into pL0016-TetR to replace the *ssurrna* fragment resulting in pL0016-TetR-3’arm. The PbEF1 promoter that was modified to contain tandem repeats of two TetO sequences at the transcription start site was synthesised (GenScript, Nanjing, China), and then inserted into PMD 18-T simple vector (Takara, Shiga, Japan) to create pT-PbEF1–2TetO plasmid. The fragment of 2A (Additional file 1: Table S1) with *BamHI*, *EcoRI* restriction site at 5′ end and *BglII*, *XhoI* at 3′ end was synthesised (GenScript), and introduced into the pT-PbEF1–2TetO plasmid to produce pT-PbEF1–2TetO-2A plasmid. Subsequently, the *gfp* fragment was amplified from pL0016 using the primers 2aGFP-1/2aGFP-717 with *BglII* and *XhoI* site, and inserted into pT-PbEF1–2TetO-2A to obtain pT-PbEF1-TetO-2A-GFP plasmid. The 5’UTR of *PblplA1* amplified using primers 5’A41/5’A42 was cloned into pT-PbEF1-3TetO-2A-GFP plasmid to create pT-PbEF1-TetO-2A-GFP-5’arm plasmid. The *lpLA1* open reading frame (*PblipL1*, PBANKA_1413000, http://plasmodb.org) was obtained from *P. berghei* cDNA by reverse transcription-PCR from *P. berghei* total RNA using the oligonucleotides LplA1–1/LplA1–2 with *BamHI* and *EcoRI* restriction site but without an endogenous stop codon. This amplified fragment was sub-cloned into pT-PbEF1-TetO-2A-GFP-5′ arm plasmid to generate pT-PbEF1–2TetO-LplA1–2A-GFP-5’UTR plasmid. This plasmid was subsequently inserted into pL0016-TetR-3’arm using *SapI* and *HindIII* restriction sites to generate a conditional knockout plasmid vector pATcon-LplA1-cKO. A control vector, pCTL-LplA1, was also constructed that contains all elements of pATcon-LplA1-cKO but lacks TetO sequences. All these transfected fragments were verified by sequencing using 3730xl DNA Analyser (ThermoFisher, Massachusetts, USA).

### Parasite in vitro culture and transfection

Blood was collected from *P. berghei-*infected BALB/c mice with heparin sodium 3–4 days post-infection when the parasitemia reached 1–3% and passed through a CF11 cellulose column (Whatman, Maidstone, UK) to remove leukocytes and platelets. The total RBCs were washed twice with RPMI-1640 medium (HyClone, Beijing, China), loaded on 74% Percoll (Sigma-Aldrich) and centrifuged at 5000× *g* for 20 min at 20 °C. The layer containing pRBCs was collected, washed twice with complete RPMI-1640 medium containing 20% FBS (HyClone), 25 mM HEPES (Sigma-Aldrich), 2 mM glutamine, 2 mg/ml glucose, 10 μg/ml hypoxanthine (Sigma-Aldrich) and 50 mg/ml neomycin sulphate (Invitrogen) and resuspended in complete RPMI-1640 medium to 2 × 10^7^/ml. The pRBCs was cultured in 6-well culture plate in a candle jar at 37 °C for 16 h [26]. During the overnight culture, most parasites developed into schizont stage.

For transfection, the pATcon-LplA1-cKO construct and the control vector pCTL-LplA1 were digested with *NotI*. The schizont-stage parasites were collected by centrifugation at 200×*g* for 5 min. pRBCs (1 × 10^7^) were resuspended with 100 μl Nucleofector Transfection Solution (Lonza, Basal, Switzerland) and the 10 mg linearized plasmid was added. The mixture was transfected by electroporation in the Nucleofector device (Lonza, Cologne, Germany) using the recommended program U-033. Fifty ml complete medium was added immediately after transfection, and the 150 μl mixture was intravenously (i.v.) injected into BALB/c mice. Forty-eight hours after injection, the mice were given 0.07 mg/ml pyrimethamine (Sigma-Aldrich) in drinking water for 7–10 days until the parasitemia reached 3–4%, the drug-resistant transgenic parasites were harvested and GFP-positive pRBCs were enriched by flow cytometer (FACS Aria II, BD Biosciences, San Jose, CA, USA) and stored in liquid nitrogen.

### Cloning of transgenic parasites

Splenectomised BALB/c mice were rested for 10 days after surgery and then injected i.v. with 200 μl per mouse clodronate liposomes (Vrije Universiteit, The Netherlands) to deplete macrophages. The enriched GFP-positive parasites were first propagated in normal BALB/c mice. The pRBCs were collected, washed with RPMI-1640 medium and the concentration of transgenic parasites was adjusted by limiting dilution to 0.7 pRBC/100 μl. One hundred μl of parasite suspension was i.v. injected into splenectomized BALB/c mice 3 days after macrophage depletion. These mice were then given ATc-containing water (0.2 mg/ml). Positive clones were detected 7–8 days after infection.

### Genotype analysis of *P. berghei*

**Fig. 1:**
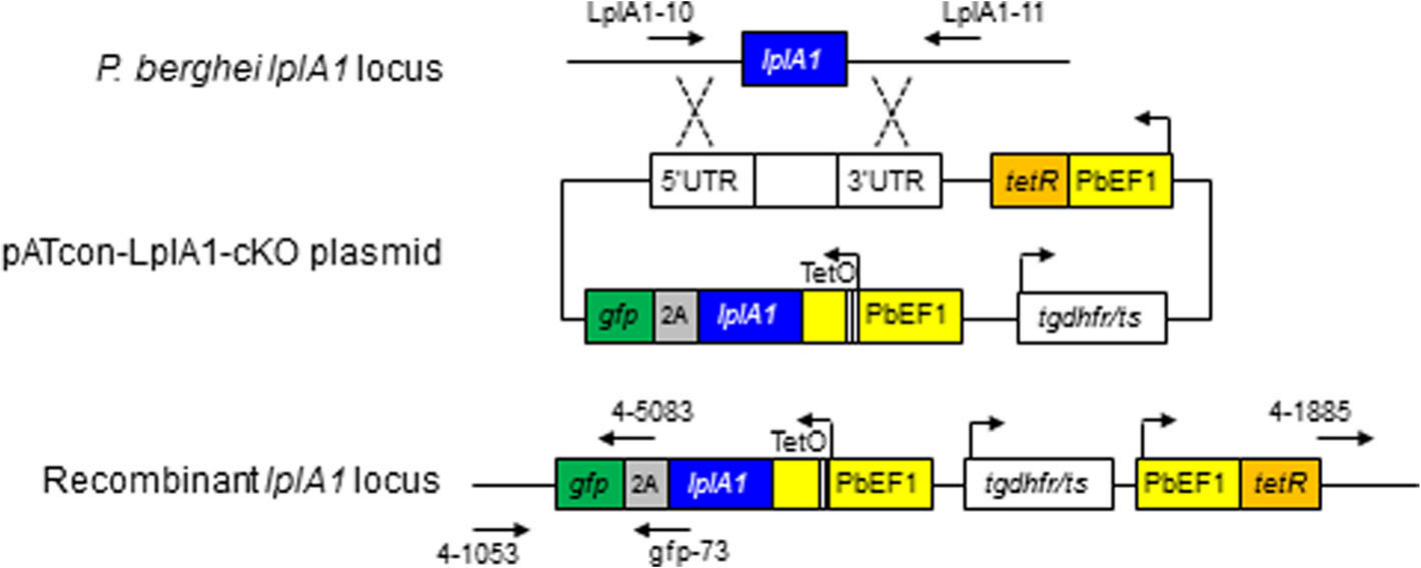
Schematic illustration of *lplA1* wild-type locus, the pATcon-LplA1-cKO construct for transfection and the recombinant locus with the *lplA1* locus replaced by double cross-over homologous recombination. Primers for PCR verification are indicated

Parasites were isolated from erythrocytes by saponin lysis as described by others [27]. Genomic DNA of transgenic and wild type parasites was obtained using DNeasy Blood & Tissue Kit (Qiagen, Hilden, Germany). Integration of the vector into parasite genome was analysed by PCR using primers (Additional file 1: Table S1; Fig. 1). The nucleotide sequences were verified by sequencing using ABI 3730xl DNA analyser (Guangzhou IGE Biotechnology, Guangzhou, China).

### Fluorescence microscopy

The enriched pRBCs were incubated for 10 min in RPMI-1640 medium containing 5 μg/ml Hoechst 33,258 (Sigma-Aldrich) at 37 °C and washed twice with the medium. The samples were applied to glass slides and imaged immediately by Zeiss 710 NLO fluorescence microscope.

### Detection of *lplA1* and *lplA2* mRNA expression by quantitative real-time PCR

Total RNA was extracted parasites using Trizol reagent (Invitrogen). Genomic DNA was removed and reverse transcription-PCR was performed with the PrimeScript™ RT Reagent Kit with gDNA Eraser (Takara), according to the manufacturer’s instruction. Quantitative real-time PCR assay was performed using the SYBR® Premix Ex *Taq*™II (Takara). The oligonucleotides LpLA1-427F/LpLA1-482R and LpLA2-572F/LpLA2-655R were used. mRNA expression of *lplA1* and *lplA2* (PBANKA_0824500) were evaluated with actin (PBANKA_145930) as a housekeeping gene using the primer actin-430F/actin-546R (Additional file 1: Table S1). The relative mRNA levels of *lplA1* and *lplA2* were calculated using the 2^-ΔΔCT^ method [28].

### Western blot analyses

Parasite proteins were extracted using RIPA lysis buffer (Beyotime, Shanghai, China) with protease inhibitor (Sigma-Aldrich) and sonicated in an ice bath (Scientz, Ningbo, China). Protein concentration was determined by BCA assay (ThermoFisher). Parasite proteins (8 μg) were separated on 10% sodium dodecyl sulphate polyacrylamide gel and then blotted onto Immobilon-P transfer membranes (Millipore, MA, USA). Membranes were incubated with a rabbit polyclonal antibody (1:10,000, Calbiochem, MA, USA) that recognizes lipoic acid attached to proteins to detect lipoylated PDH, KGDH and BCDH, mouse monoclonal antibody (mAb) specific for TetR protein (1:1000, Clontech, CA, USA) or mouse mAb to β-actin as a loading control (1:1000, Sigma-Aldrich). Sheep anti-rabbit or rabbit anti-mouse antibodies conjugated with horseradish peroxidase (1:4000) were used as secondary antibodies for detection with Supersignal West Pico Chemiluminescence Kit (ThermoFisher).

### Parasite growth in vitro and in vivo

To detect whether ATc regulates the blood stage growth of transgenic parasites in vivo, groups of mice were infected with 1 × 10^6^ pRBC of wild-type or transgenic *P. berghei* and provided with normal drinking water or water containing 0.2 mg/ml ATc [29]. Parasitemia was monitored daily by examination of Giemsa-stained thin blood smears.

For in vitro analysis, blood was collected from the mice at early (day 6) and late (day 14) stage of infection. Blood samples were passed through the CF11 column and centrifuged on 74% Percoll at 5000× *g* for 20 min at 20 °C. pRBC layer was collected, washed twice with complete medium, and incubated in complete medium with or without 1 μg/ml ATc for 16 h in a candle jar. The cultured parasites were collected, and thin smears were made on the glass slides stained by Giemsa-solution and analysed by microscopy.

### Statistical analysis

All infection experiments were performed three times, and data from one of them are presented. Each experimental group consists of three to five mice and mean (± SD) are shown. Student *t*-test was performed to determine the significance of difference between experimental groups using GraphPad Prism (GraphPad Software Inc., CA, USA).

## Results

### Generation of *lplA1* conditional knockout (LplA1-cKO) parasites

**Fig. 2:**
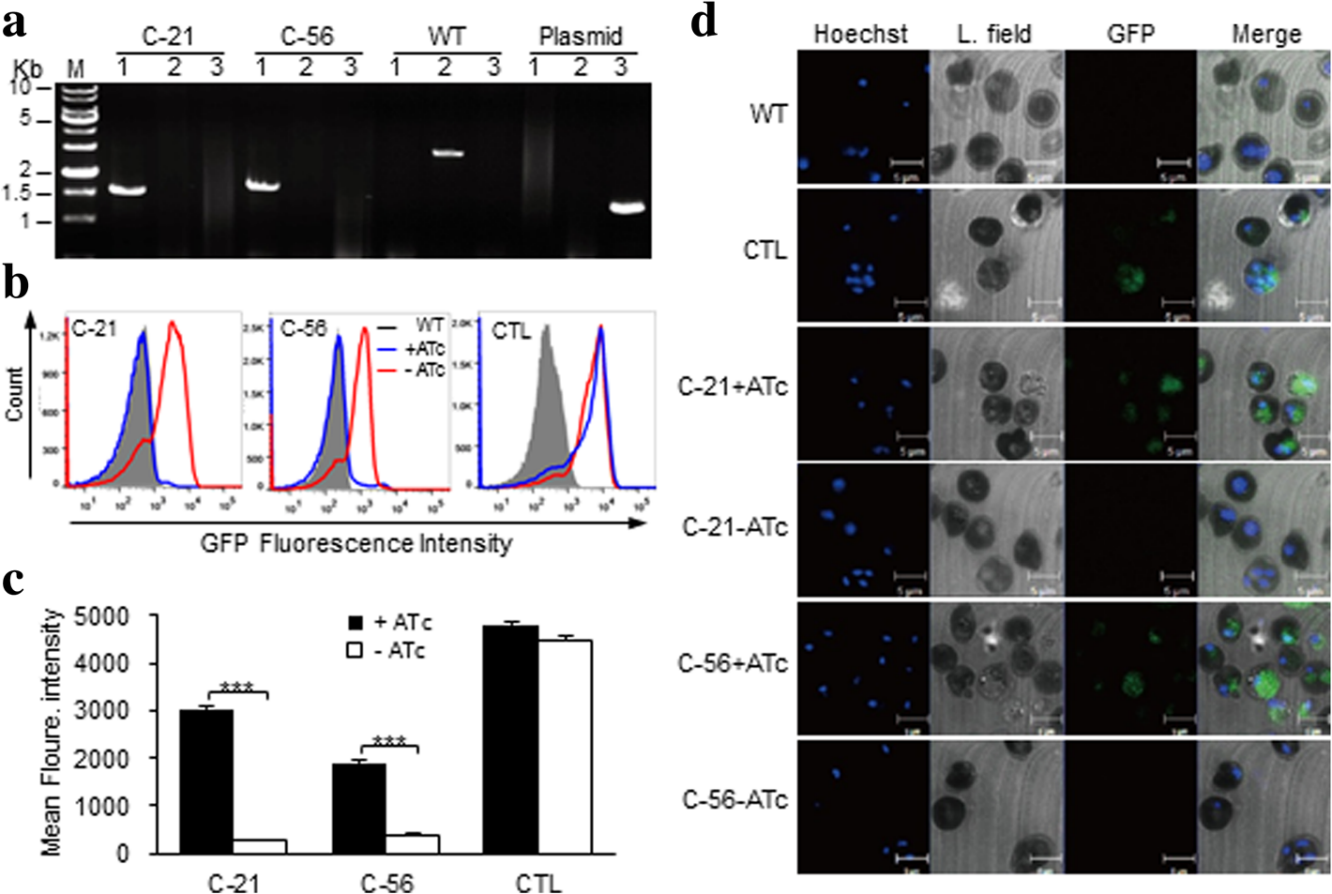
Genotype confirmation and GFP expression profile of clone 21 (C-21) and 56 (C-56) of LplA1-cKO transgenic parasites. **a** Genotype of cloned transgenic parasites were analyzed by PCR using the primer pairs gfp-73/4–1053 (Lane 1) to detect a 1.6 kb fragment crossing parasite genome and the vector, LplA1–10/LplA1–11 (Lane 2) to amplify a 2.5 kb fragment crossing *lplA1* locus in WT parasite, and 4–1885/4–5083 (Lane 3) to amplify a 0.99 kb fragment of the plasmid vector or episome (also see legend to Fig. 1). The fragments were verified by sequencing. **b** Flow cytometric profiles of *gfp* expression by cloned LplA1-cKO or control transgenic parasites with or without exposure in vivo to ATc. **c** Mean GFP fluorescence intensity of cloned LplA1-cKO parasites. Mean (± SD) from triplicate flow cytometric analyses are presented, ***P < 0.001 (Student’s *t*-test). **d** Fluorescence photomicrographs showing *gfp* expression by transgenic parasites with or without ATc exposure in vivo. *Scale-bars*: 5 μm

We developed an ATc-regulated expression system for blood-stage *P. berghei* that contains a regulatory unit expressing the repressor protein TetR controlled by the PbEF1 promoter and a responsive unit consisting of the modified PbEF1 to control the expression of the target gene as well as the *gfp* reporter. The PbEF1 promoter was modified by insertion of two TetO sequences at the transcription start sites (TSS) that were determined by 5’RACE and nested PCR. The expressed TetR binds to the TetO sequences to inactivate the PbEF1 promoter. In the presence of ATc, the TetR dissociates from TetO and PbEF1 resumes the activity to drive the target gene expression (ATc-on system). Using this system, we constructed a plasmid vector, pATcon-LplA1-cKO, to conditionally knockout *lplA1* by double cross-over homologous recombination. Blood-stage *P.berghei* parasites were transfected with the vector, and the drug-resistant population was obtained. The pRBC was cloned by limiting dilution and injection into splenectomized and macrophage-depleted BALB/c mice. Parasitemia emerged in 12 of 60 cloning mice. Analysis of genotype of the 12 clones by PCR revealed that one clone showed mixed genotype (data not shown), and the other 11 clones showed the correct integration of the vector into parasite genome, and no episome was detected (Additional file 1: Figure S1a; Fig. 2a). In addition, control parasites that carry the control vector pCTL-LplA1 were generated and cloned in the same manner. To determine the regulatory activity of ATc-on system, groups of mice were infected with the cloned transgenic parasites and were provided with normal or ATc-containing water. The pRBCs were collected 6 days after infection and analysed by flow cytometry to determine *gfp* reporter expression. It was observed that, although the 11 parasite clones showed different *gfp* expression profiles, the parasites exposed to ATc in vivo expressed higher levels of GFP compared with the parasites of the same clone that was not exposed to ATc. As expected, the parasites transfected with control vector pCTL-LplA1 expressed a high level of GFP regardless of ATc exposure (Additional file 1: Figure S1b, S1c; Fig. 2b, c). Two clones, clone 21 and 56 that showed greatest difference in *gfp* expression with or without ATc treatment (Student’s *t*-test: clone 21, *t*_(4)_ = 18.10, *P* < 0.001; clone 56, *t*_(4)_ = 15.30, *P* < 0.001; Fig. 2c), were selected for further analysis. It was also observed by microscopy that the parasites of clone 21 and 56 exposed to ATc in vivo expressed abundant GFP that was absent in the parasites without exposure to ATc (Fig. 2d).

### Phenotypes of LplA1-cKO parasites

**Fig. 3:**
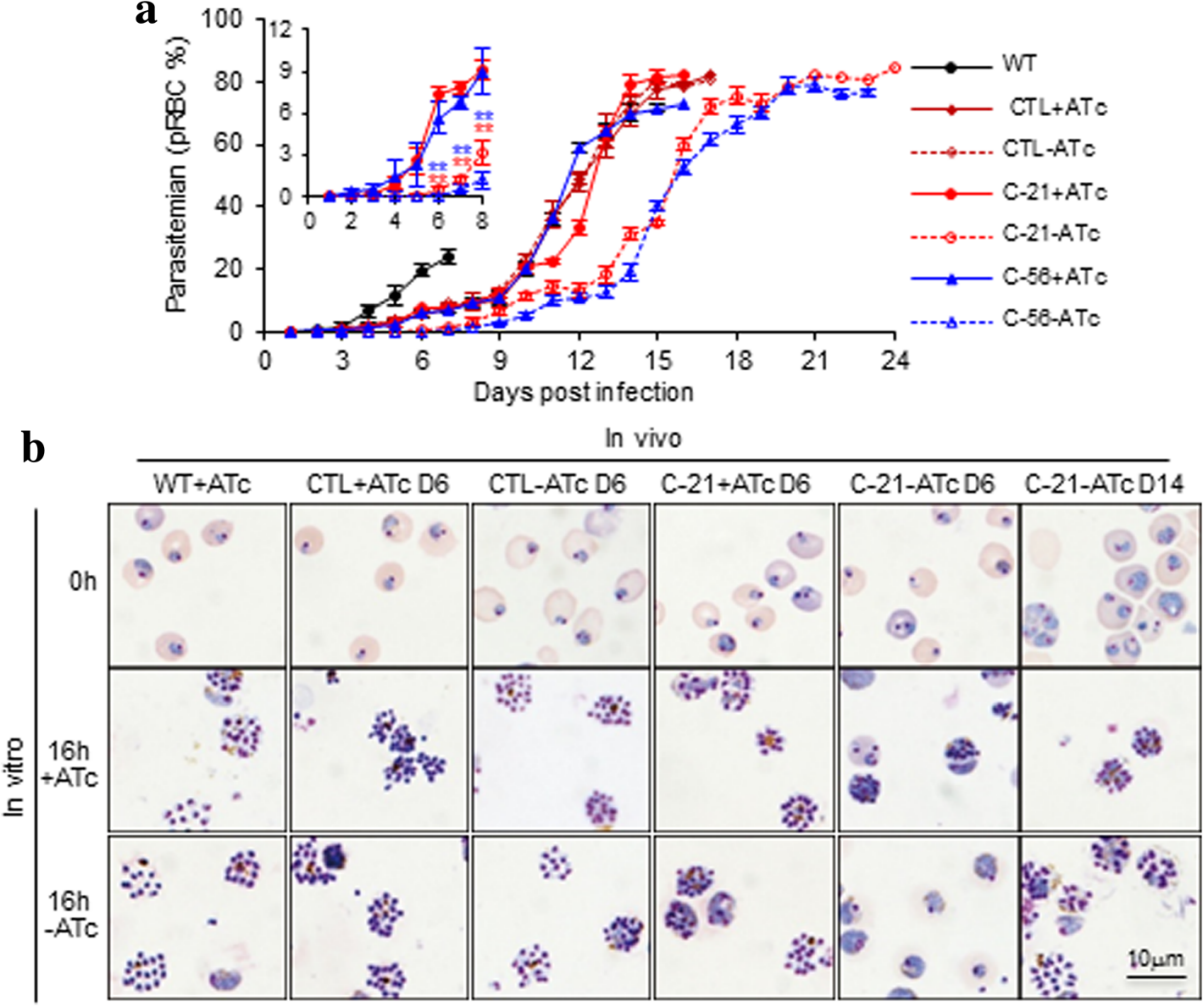
Proliferation in vivo and development in vitro profiles of cloned LplA1-cKO parasites in the presence or absence of ATc. **a** Parasitemia levels in mice infected with cloned LplA1-cKO parasites and provided with normal or ATc-containing water. Results shown are from one of two experiments. Data are mean (± SD) of 3–5 mice per group. The insert shows the parasitemia during the first 8 days of infection. **P < 0.01 (Student’s *t*-test) compared to parasites of the same clone exposed to ATc. **b** In vitro development of LplA1-cKO parasites collected in the early (D6) and late (D14) stage of infection. Photomicrographs of clone 21 (C-21) parasites are shown. *Scale-bar*: 10 μm

To determine whether repressed expression of *lplA1* affects the viability of the parasite, groups of mice were infected with Clone 21 and 56 transgenic parasites, as well as the control vector-transfected parasites, provided with normal or ATc-containing drinking water. Parasite growth was monitored daily. The mice infected with parasites of clone 21 and 56 and provided with ATc developed high levels of parasitemia and succumbed to day 15–17 post-infection. The mouse groups infected with the same clones of transgenic parasites and given normal water showed significantly lower levels of parasitemia in the early stage (up to day 8) of infection compared with the same clone of parasite exposed to ATc (Student’s *t*-test: clone 21, day 6, *t*_(5)_ = 14.21, *P* < 0.001; day 7, *t*_(5)_ = 13.93, *P* < 0.001; day 8, *t*_(5)_ = 8.01, *P* < 0.01; clone 56, day 6, *t*_(5)_ = 12.12, *P* < 0.001; day 7, *t*_(5)_ = 14.11, *P* < 0.001; day 8, *t*_(5)_ = 9.11, *P* < 0.01; Fig. 3a insert). However, in the late stage of infection, the LplA1-cKO transgenic parasites resumed the proliferation ability and high levels of parasitemia developed in the mice (Fig. 3a). It was also noticed that the LplA1-cKO and control transgenic parasites showed slower growth in the presence of ATc when they were compared with the WT parasites (Fig. 3a).

We then performed an experiment to examine the development and differentiation of the transgenic parasites in vitro. pRBCs were collected from the mice at early (day 6) or late (day 14) stages of infection and cultured in vitro for 16 h in the presence or absence of ATc. The LplA1-cKO parasites of clone 21 exposed to ATc in vivo collected at day 6 of infection developed from early ring stage to schizont stage in vitro in the presence or absence of ATc. The same transgenic parasites that were not exposed to ATc in vivo and collected at day 6 of infection were not able to develop to schizont stage in vitro in the absence of ATc. However, the LplA1-cKO parasites that were not exposed to ATc in vivo and collected at day 14 showed normal development from ring to schizont stage in vitro when ATc were not provided (Fig. 3b). These results demonstrate that the LplA1-cKO parasites could resume their viability in the absence of ATc in the late stage of infection.

### Redundant pathway of lipoic acid metabolism

**Fig. 4:**
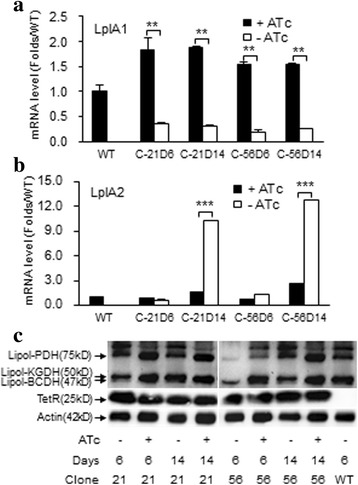
Levels of *lplA1* and *lplA2* mRNA expression and lipoylated KADHs by cloned LplA1-cKO parasites collected in early and late stage infection. **a** *lplA1* mRNA levels determined by quantitative PCR. Data are mean ± SD from three analyses. **P < 0.01 (Student’s *t*-test). **b** *lplA2* mRNA levels determined by quantitative PCR. ***P < 0.001 (Student’s *t*-test). **c** Levels of lipoylated PDH, KGDH and BCDH were analysed by protein blotting using specific antibodies. TetR protein levels were analyzed in parallel. Actin was used for control of sample loading

The observation that LplA1-cKO parasites resumed the proliferation ability in the late stage of infection in the absence of ATc suggests the existence of a redundant pathway for lipoic acid metabolism. We collected the parasites at early (day 6) and late (day 14) stages of infection and determined mRNA expression of *lplA1* and *lplA2* genes by quantitative PCR. The parasites exposed to ATc in vivo showed *lplA1* mRNA levels that were higher than that of wild-type parasites. The levels of *lplA1* mRNA expressed by the parasites that were not exposed to ATc in vivo and collected at day 6 of infection were significantly lower than the levels of ATc-exposed transgenic parasites (Student’s *t*-test: clone 21, *t*_(4)_ = 4.99, *P* < 0.01; clone 56, *t*_(4)_ = 8.77, *P* < 0.01; Fig. 4a) and the wild-type parasites. At the late stage (day 14) of infection, the transgenic parasites that were not exposed to ATc still expressed lower levels of the *lplA1* mRNA compared with the parasites exposed to AT (Student’s *t*-test: clone 21, *t*_(4)_ = 10.34, *P* < 0.01; clone 56, *t*_(4)_ = 8.45, *P* < 0.01; Fig. 4a), indicating that the *lplA1* gene expression is under the control of Tet-on system in the course of infection. The pattern of *lplA2* mRNA expression was different from that of *lplA1*. The parasites at an early stage of infection showed minimum expression of *lplA2* mRNA. However, at the late stage of infection the parasites that were not exposed to ATc produced remarkably higher levels of *lplA2* mRNA which was not observed in the parasites exposed to ATc (Student’s *t*-test: clone 21, *t*_(4)_ = 38.39, *P* < 0.001; clone 56, *t*_(4)_ = 92.98, *P* < 0.001; Fig. 4b). We then determined lipoylation of the α-keto acid dehydrogenases (KADH) in the transgenic parasites from early and late stage of infection by immune blotting. At the early stage (day 6) of infection, the transgenic parasites that were not exposed to ATc showed reduced levels of lipoylated KGDH and BCDH in both clone 21 and 56 and reduced PDH in clone 56 than the parasites exposed to ATc. However, these differences in the levels of lipoylated KADH were not detected between parasites with and without ATc exposure at the late stage of infection (Fig. 4c). It has been reported by others that H-protein is not abundant in the parasites [18, 19]. We were unable to detect the lipoylated H-protein in the protein blotting with the polyclonal antibody used in this study (data not shown). In addition, no difference in TetR protein expression was detected in parasites at early and late of infection (Fig. 4c).

## Discussion

LA-dependent multienzyme complexes are involved in energy metabolism. The enzymes that catalyse the synthesis of LA or ligation of LA to the multienzyme complexes, therefore, are critical for the survival of the organisms. Previous studies in apicomplexan *Plasmodium* parasites have revealed many unique features of the LA-related metabolism machinery that offer the opportunity for identification of potential targets for intervention of malaria [13, 14, 16, 30]. It has been shown that LplA1 plays important roles in salvage of LA from the environment and ligation of LA to KADH in the mitochondrion in *P. falciparum* parasites [18]. A gene deletion study in *P. berghei* also showed that this gene is essential for the survival of the parasite [21]. In the present study, we demonstrate that conditionally switching-off *lplA1* gene expression resulted in significant impairment of the proliferative ability of the transgenic *P. berghei* parasites in the early phase (first 8 days) of blood-stage infection. This result indicates that LplA1 is critically required for the survival of parasites. However, the transgenic parasites in the absence of ATc regained the viability in the late stage of infection and developed high levels of parasitemia leading to the death of the hosts. Loss of the defective phenotype of the transgenic parasites in the late stage of infection is not due to the malfunctioning of the cKO vector because the parasites collected at this stage of infection were able to produce the TetR repressor protein (Fig. 4c), and the *lplA1* mRNA expression was efficiently regulated by ATc (Fig. 4a). Further examination showed that the LplA1-cKO parasites that were not exposed to ATc and collected in the late stage of infection expressed a high level of *lplA2* mRNA which was not observed in the transgenic parasites exposed to ATc. We propose here that LplA2 might be a redundant mechanism that is induced and compensates for the loss of LplA1 activity to maintain the critical LA metabolism pathway for the survival of the parasites. Lipoylation of PDH is known to occur mainly in the apicoplast [13, 15]. We observed that, in the absence of ATc, the transgenic parasites showed reduced levels of lipoylated PDH at an early stage of infection, but this was corrected at the late stage of infection (Fig. 4c). The increased level of lipoylated PDH observed in the late stage of infection is probably due to the increased expression of LplA2 that is present in both mitochondrion and apicoplast [15, 19]. In this study, we did not examine the activity of LipB and LipA in the LplA1-cKO transgenic parasite and cannot rule out the roles of these two enzymes in compensation for the loss of LplA1 activity.

The Tet-on gene expression system established and used here in blood-stage *P. berghei* parasites showed an efficient regulatory activity. We observed that in the presence of ATc, the transgenic parasites expressed *lplA1* mRNA level that was 1.5–1.8-fold higher than the level detected in WT parasites. This is possibly due to the PbEF1 that is known to be highly active in all development stages of the parasite [25]. The possible effect of over-expression of *lplA1* gene on the parasite biology was not evaluated in detail. However, we observed that the LplA1-cKO and CTL-cKO parasites treated with ATc showed differences in the growth profile in comparison with WT *P. berghei*. Unlike the WT parasites that developed rapidly and caused the death of host due to cerebral malaria early during infection, the transgenic parasites with the treatment of ATc proliferated in lower rates and eventually reached severe parasitemia, and mice died in the late stage of infection due to anaemia.

Günther et al. [21] have demonstrated the critical role of LplA1 for the viability of *P. berghei* because permanent disruption of this gene impeded the parasite survival. We observed in our study that the LplA1-cKO parasites with no ATc treatment showed severely impaired proliferation ability in the first 8 days of infection, evidence to support the important role of LplA1 for parasite growth. The low level of growth seen in the transgenic parasites is attributable to the residual level of LplA1 resulting from the leaky expression of the Tet-on vector in the absence of ATc. Under this stressful condition, the surviving parasites may activate LplA2 redundancy pathway for LA metabolism to support the parasite growth.

## Conclusions

We used a conditional gene knockout approach to investigate the role of *lplA1* gene for the survival of blood-stage *P. berghei* parasite. Our results revealed that LplA1 is required for the growth of the parasite in normal condition. However, the *lplA2* expression can be activated as a redundancy pathway to compensate for the loss of LplA1 function. We also demonstrated that the ATc-regulated gene expression system is a valuable tool not only for the investigation of gene function but also for analysis of phenotypic changes and determination of potential alternative pathway(s) of the genetically modified malaria parasites.

## Additional file

Additional file 1: Figure S1. Genotype and phenotype analysis of LplA1-cKO transgenic parasite clones. **a** PCR verification of genotypes of the LplA1-cKO parasite clones. **b** Regulation of GFP expression by ATc in cloned LplA1-cKO parasites. **c** Mean GFP fluorescence intensity of 9 LplA1-cKO clones. Each clone was analysed by flow cytometry in triplicate, and the mean + SD was reported. **Table S1**. Nucleotide sequences of primers and the 2A peptide used in the study. (DOCX 678 kb)

## Abbreviations

ATc: Anhydrotetracycline
BCDH: Branched chain-ketoacid dehydrogenase
FACS: Fluorescence-activated cell sorting
GCS: Glycine cleavage system
KGDH: α-ketoglutarate dehydrogenase
LA: Lipoic acid
LipA: Lipoic acid synthase
LipB: Octanoyl-[aycl carrier protein]: protein N-octanoyl transferase
LplA1: Lipoic acid protein ligase 1
LplA2: Lipoic acid protein ligase 2
PDH: Pyruvate dehydrogenase
