# Supplementary material for: Conditional knock-out of lipoic acid protein ligase 1 reveals redundancy pathway for lipoic acid metabolism in Plasmodium berghei malaria parasite
Source: Parasit Vectors. 2017 Jun 27;10:315. doi: 10.1186/s13071-017-2253-y (PMC5488443; doi:10.1186/s13071-017-2253-y)
Supplement: Additional file 1: Figure S1. — Genotype and phenotype analysis of LplA1-cKO transgenic parasite clones. a PCR verification of genotypes of the LplA1-cKO parasite clones. b Regulation of GFP expression by ATc in cloned LplA1-cKO parasites. c Mean GFP fluorescence intensity of 9 LplA1-cKO clones. Each clone was analysed by flow cytometry in triplicate, and the mean + SD was reported. Table S1. Nucleotide sequences of primers and the 2A peptide used in the study. (DOCX 678 kb) [file 13071_2017_2253_MOESM1_ESM.docx]

**Additional file**

Clone 14


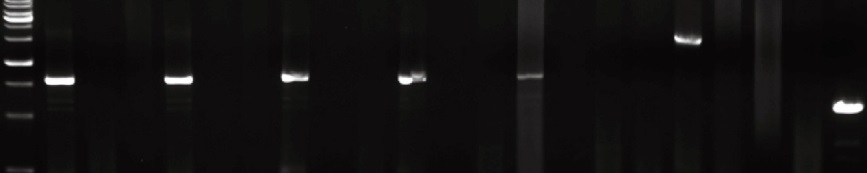


1

2

3

1

2

3

1

2

3

1

2

3

1

2

3

M

Plasmid

1

2

3

1

2

3

WT

22

15

24

26

Clone 34

CTL

36

52

53

WT

Plasmid


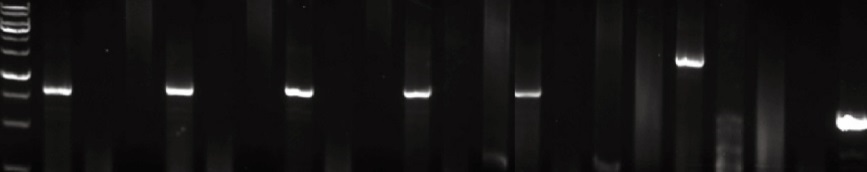


1

2

3

M

1

2

3

1

2

3

1

2

3

1

2

3

1

2

3

1

2

3

1.5

5

2

1

10

kb

1.5

5

2

1

10

kb

a

GFP Fluorescence intensity

Cell counts

WT

+ ATc

- ATc


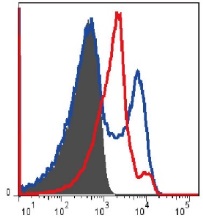

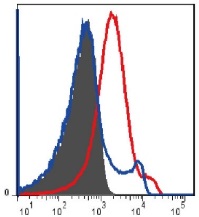

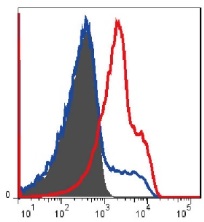

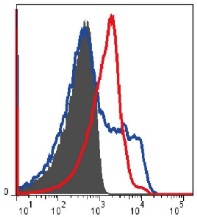

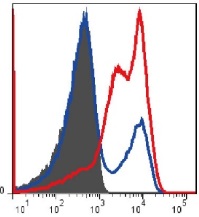


C-14

C-15

C-22

C-24

C-26


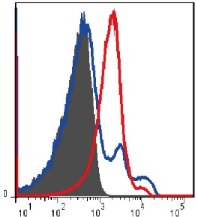

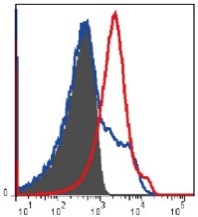

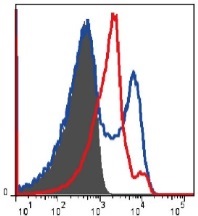

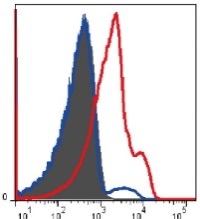

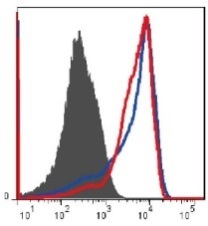


C-34

C-36

C-52

C-53

CTL

b

c


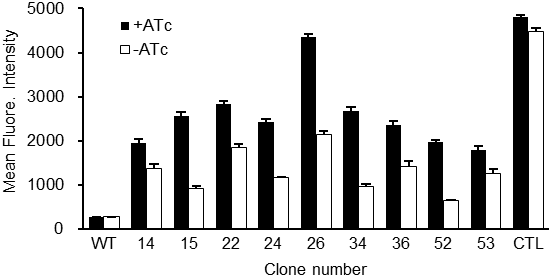


Figure S1. Genotype and phenotype analysis of LplA1-cKO transgenic parasite clones. **a**. Genomic DNA was isolated from the cloned transgenic and wild-type parasite and analyzed by PCR using primer pairs. Lane 1: gfp-73/4-1053 to amplify a 1.6 kb fragment crossing the parasite genome and the vector; lane 2: LplA1-10/LplA1-11 to amplify a 2.5 kb fragment crossing LplA1 locus in WT parasite; lane 3: 4-1885/4-5083 to amplify a 0.99 kb fragment of the plasmid vector or episome. **b**. LplA1-cKO parasite and control vector transfected parasite clones were collected from mice that were given normal or ATc-containing water and analyzed for GFP expression by flow cytometry. WT parasites were included as negative control. **c**. Mean GFP fluorescence intensity of 9 LplA1-cKO clones and the control vector parasite clone. Each clone was analyzed by flow cytometry in triplicate and the mean+SD was reported.

Table S1. Nucleotide sequences of primers and the 2A peptide used in the study

|  | Sequences (5’-3’) | Restriction site |
| --- | --- | --- |
| 2A | AAAATTGTCGCTCCTGTCAAACAAACTCTTAACTTTGATTTACTCAAACTGGCTGGGGATGTAGAAAGCAATCCAGGTCCA |  |
| TetR-1 | CGC*GGATCC*ATGTCCAGATTAGATAAAAGTAAAGTGATTAACAG | *BamHI* |
| TetR-657 | AGT*GGATCC*TTAATAAGATCTGAATTCCCGAGATCCGCTGTACGCGGAC | *BamHI* |
| 2aGFP-1 | CGG*AGATCT*ATGAGTAAAGGAGAAGAAC | *BglII* |
| 2aGFP-717 | ACA*CTCGAGGATATC*TTATTTGTATAGTTCATCCATG | *XhoI, EcoRV* |
| 3'A41 | CCG*CTCGAG*CCAATACTTTAAAACATTTAACAATC | *XhoI* |
| 3'A42 | TAT*GAATTCGCGGCCGC*GGACAAGCATAGCTTATGCCCGATC | *EcoRI, NotI* |
| 5'A41 | AGCTTT*GTTTAAACGCGGCCGC*TACTATATATTTAATATATAACAGGG | *PmeI, NotI* |
| 5'A42 | CTA*TCTAGA*CCAACGATAAATTAAAGTAAATATTTTG | *XbaI* |
| LplA1-1 | CGC*GGATCC*ATGCCAGGTATATCCTGTTTT | *BamHI* |
| LplA1-2 | CCG*GAATTC*GAGTTCTTCTAATATCCATGAAG | *EcoRI* |
| LplA1-10 | GGATAATGTAATAAAATCTAGCCATTTAACTC |  |
| LplA1-11 | GCGCGTGTTGGTGTGTATATGAGAAATTCC |  |
| 4-1053 | CAATCATTCTCTAAGGGGCGTATAGT |  |
| gfp-73 | GAGACCACATGGTCCTTCTTGAGTT |  |
| 4-1885 | ACGACCTCAAAAGAGAGCAAATTAT |  |
| 4-5083 | ACTAGTTATAACCCTTTGGAGAAA |  |
| actin-430F | TATTCATCAGGCCGTACAACAGG |  |
| actin-546R | AGCCAAATCTAATCTCATTATTGCAT |  |
| LPLA1-427F | AACATTGATGCAAAATTACATGGAAG |  |
| LPLA1-482R | ATATTTTTAAATGCAGATCCTGAGC |  |
| LPLA2-572F | AAACTTTTCAATATCACGAAC |  |
| LPLA2-655R | CTTGTGCATTTCCTCCAACTTT |  |
